# Supplementary material for: Impact of intensifying nitrogen limitation on ocean net primary production is fingerprinted by nitrogen isotopes
Source: Nat Commun. 2021 Oct 28;12:6214. doi: 10.1038/s41467-021-26552-w (PMC8553784; doi:10.1038/s41467-021-26552-w)
Supplement: Supplementary file 1 — Supplementary Information [file 41467_2021_26552_MOESM1_ESM.pdf]

Supplementary material to: Impact of intensifying nitrogen limitation on ocean net primary production is fingerprinted by nitrogen isotopes

Pearse J. Buchanan[1*], Olivier Aumont[2], Laurent Bopp[3], Claire Mahaffey[1], and Alessandro Tagliabue[1].

[1]Department of Earth, Ocean and Ecological Sciences, University of Liverpool, Liverpool, United Kingdom.
[2]Laboratoire d'Océanographie et du Climat: Expérimentations et Approches Numériques (LOCEAN), IPSL, Sorbonne Université, IRD, CNRS, MNHN, Paris, France.
[3]Laboratoire de Météorologie Dynamique (LMD), IPSL, Ecole Normale Supérieure - Université PSL, Sorbonne Université, Ecole Polytechnique, CNRS, Paris, France

*Corresponding author: pearse.buchanan@liverpool.ac.uk

Supplementary Information

- Supplementary Note 1 – Nitrogen isotope routines
- Supplementary Note 2 – Nitrogen isotope assessment ($\delta^{15}N_{NO3}$)
- Supplementary Note 3 – 0D model dynamics
- Supplementary Figures
- Supplementary Tables
- Supplementary References

Supplementary Material to: Impact of intensifying nitrogen limitation on ocean net primary production is fingerprinted by nitrogen isotopes

Supplementary Note 1

The nitrogen isotopic routines carry ^{15}N through nitrate (NO_3), ammonium (NH_4), both phytoplankton types, both zooplankton types, small and large particulate organic matter, as well as dissolved organic matter.

The isotopic signature of nitrogen is expressed in delta notation in units of per mil ‰, where:

$$\delta^{15}N = \left(\frac{\left(\frac{^{15}\mathrm{N}}{^{14}\mathrm{N}}\right)^{sample}}{\left(\frac{^{15}\mathrm{N}}{^{14}\mathrm{N}}\right)^{standard}} - 1 \right) \cdot 1000 \quad (1)$$

$$\left(\frac{^{15}\mathrm{N}}{^{14}\mathrm{N}}\right)^{standard} = 0.003676 \quad (2)$$

Key fractionation processes take place during NO_3 and NH_4 assimilation by phytoplankton, denitrification in both the sediments and water column, ingestion of prey by zooplankton, and excretion of NH_4 by zooplankton.

Key sources of ^{15}N are atmospheric reactive nitrogen (N_r) deposition, riverine dissolved inorganic nitrogen, and organic nitrogen fixed by diazotrophs.

All fractionation processes of nitrogen isotopes are biologically mediated and all follow the same formula. Here, we illustrate the formula of nitrogen isotope fractionation using phytoplankton assimilation of NO_3 into biomass during primary production.

$$^{15}N_{t+1}^{phy} = {}^{15}N_t^{phy} + NO_3^{assimilated} \cdot \left(\frac{^{15}\mathrm{N}}{^{14}\mathrm{N}}\right)^{NO_3} \cdot \alpha_{phy} \quad (3)$$

$$^{15}N_{t+1}^{NO_3} = {}^{15}N_t^{NO_3} - NO_3^{assimilated} \cdot \left(\frac{^{15}\mathrm{N}}{^{14}\mathrm{N}}\right)^{NO_3} \cdot \alpha_{phy} \quad (4)$$

For each reaction in the nitrogen cycle involving fractionation, the fractionation factor (α) has been experimentally determined by measuring the isotopic ratios of the products and reactants, in this case:

$$\alpha = \left(\frac{\left(\frac{^{15}\mathrm{N}}{^{14}\mathrm{N}}\right)^{product}}{\left(\frac{^{15}\mathrm{N}}{^{14}\mathrm{N}}\right)^{reactant}} \right) \quad (5)$$

Isotope fractionation factors are close to one and so are often represented in per mil units using the ε notation, where:

$$\varepsilon = (1 - \alpha) * 1000 \quad (6)$$

The fractionation factors for most reactions are assumed constant, with the exception of NO_3 and NH_4 assimilation by phytoplankton. Their default values are:

- Phytoplankton assimilation (α_{phy}) = 0.995 (ε_{phy} = 5 ‰)
- Water column denitrification (α_{wc}) = 0.975 (ε_{wc} = 25 ‰)
- Sedimentary denitrification (α_{sed}) = 0.997 (ε_{sed} = 3.0 ‰)
- Nitrification (α_{nit}) = 1.0 (ε_{nit} = 0.0 ‰)
- Zooplankton ingestion (α_{ing}) = 1.001 (ε_{ing} = -1.0 ‰)
- Zooplankton excretion (α_{exc}) = 0.994 (ε_{exc} = 6.0 ‰)

Here, positive ε values indicate an enrichment in ^{15}N within the reactant and a depletion in the product.

Fractionation during the assimilation of NO_3 and NH_4 by phytoplankton vary according to the ratio of demand and supply of each nutrient. If demand is high but supply is low, such that the amount of nitrogen available to phytoplankton is limiting, then α_{phy} approaches one ($\epsilon_{phy} \rightarrow 0$ ‰). If, however, NO_3 or NH_4 are in high concentrations and/or demand is low, then fractionation proceeds at its maximum value, set at $\alpha_{phy} = 0.995$ ($\epsilon_{phy} = 5.0$ ‰). This utilisation effect is calculated by dividing the NO_3 (NH_4) required by the NO_3 (NH_4) available, and is then multiplied against 5.0 ‰.

Sources of ^{15}N are atmospheric N_r deposition, riverine dissolved inorganic nitrogen, and organic nitrogen fixed by diazotrophs (nitrogen fixers). The signatures of these sources are prescribed using input files during simulations. The default $\delta^{15}N$ signatures of these sources are as follows:

- Atmospheric N_r deposition $\delta^{15}N_{DIN} = -4.0$ ‰
- Riverine $\delta^{15}N_{DIN} = 2.0$ ‰
- Diazotrophy $\delta^{15}N_{org} = -1.0$ ‰

These processes combine to form the $\delta^{15}N$ distribution of NO_3 and total particulate organic matter (POM) show in Supplementary Figure 1.

Supplementary Material to: Impact of intensifying nitrogen limitation on ocean net primary production is fingerprinted by nitrogen isotopes

Supplementary Note 2

We used a global compilation of $\delta^{15}N$ of nitrate ($\delta^{15}N_{NO3}$) measurements[1], supplemented with data from the Arctic Ocean, to compare with the model. This measurement dataset contained 13,096 measurements from all ocean basins over years 1971 to 2018. We made one-to-one comparisons with simulated $\delta^{15}N_{NO3}$ using the 1986-2005 average of the climate change experiment with anthropogenic nitrogen deposition by gridding the observations onto the model grid. If multiple measurements occurred in the same bin, these were averaged. Due to averaging and masking at model land tiles, the model-data comparison involved a total of 7,661 comparisons covering 0.7% of the model's volume.

Simulated $\delta^{15}N_{NO3}$ provided an adequate overall fit to the *in situ* $\delta^{15}N_{NO3}$ measurements (Supplementary Figures 2 and 3). A global correlation of 0.62 and regional correlations ranging from -0.38 to 0.84 revealed the difficulty in reproducing *in situ* $\delta^{15}N_{NO3}$ measurements, a difficulty shared by other global biogeochemical models[2]. The worst regional fit, with a correlation of -0.34, was for the Indian Ocean, where the model oxygen field misrepresented the oxygen minimum zones in the Arabian Sea and Bay of Bengal. Because most observations of $\delta^{15}N_{NO3}$ are from the Arabian Sea, the misplacement of the low oxygen zone had a strong effect on the regional fit. In fact, measurements programs are biased towards sampling within or near to the oxygen minimum zones where denitrification is active, but where the strongest gradients in $\delta^{15}N_{NO3}$ exist in the modern ocean. The best regional fit therefore, with a correlation of 0.84, was for the Southern Ocean where the $\delta^{15}N_{NO3}$ distribution is primarily affected by the physical positioning of water masses and summertime productivity, and the spatial gradients are weaker. All regions suffered from subdued variance compared with the data, which was evident by low normalised standard deviations, but is to be expected when comparing measurements, which are prone to high frequency variability, to monthly-averaged model output on a coarse grid.

Supplementary Note 3

Our 0-D isotope model contains three pools of nitrogen. These are dissolved inorganic nitrogen (DIN), particulate organic nitrogen (PON) and exported nitrogen (ExpN), all in the same units of mmol N m^{-3}. Initially, the DIN pool represents the bulk of total nitrogen in the model. Over time, phytoplankton consume DIN and increase the pool of PON. This subsequently increases ExpN as the rate of export is to a first order dependent on the biomass of PON (Supplementary Figure 10a).

Rates of DIN uptake [DIN→PON], PON recycling [PON→DIN] and PON export [PON→ExpN] all increase as the PON pool increases (Supplementary Figure 10b). Once DIN is depleted to a limiting concentration near zero (~0.07 mmol m^{-3}), the PON pool no longer grows. Now, the rate of DIN uptake closely matches the rate of PON recycling, such that all primary production occurs through regeneration. The PON pool begins to decline as nitrogen is lost via export.

The shift into a DIN-limited system, where primary production is regenerated rather than new, also coincides with a decrease in fractionation associated with DIN uptake (Supplementary Figure 10c). Here, the limiting concentrations of DIN mean that phytoplankton have less preference for the lighter isotope over the heavier isotope[3,4].

These dynamics result in the $\delta^{15}N$ trends shown in Supplementary Figure 10d. High rates of new production and strong fractionation initially drive an increase in $\delta^{15}N_{DIN}$, which is paralleled by $\delta^{15}N_{PON}$ at a constant offset equal to ε_{phy} (~5 ‰). As DIN is consumed towards limiting concentrations, all primary production transitions from mostly new to mostly regenerated (i.e. supported by recycling). New DIN produced through recycling has an isotopic signature equal to $\delta^{15}N_{PON}$, and due to the low concentration of remaining DIN, $\delta^{15}N_{DIN}$ approximates $\delta^{15}N_{PON}$. Thus, phytoplankton under DIN-limited conditions take on an isotopic signature close to that of the PON pool, with a constant offset equal to ε_{phy}, which under DIN-limited conditions is near zero. Over the course of the simulation, the combined DIN and PON pools have become increasingly enriched in ^{15}N due to export of PON that is relatively depleted in ^{15}N.

Under a closed system with no recycling, isotopic dynamics instead obey the Rayleigh model[5,6]. In such a case, the isotopic signature of the reactant ($\delta^{15}N_{DIN}$) would increase towards infinity as its consumed towards zero, while the isotopic signature of the product ($\delta^{15}N_{PON}$) would increase towards the initial isotopic signature of the reactant. The dynamics of our simulations differ from this closed-system model because we (i) recycle some of the product back to the reactant pool (i.e. a back-reaction), and (ii) export of some of the product out of the system.

Supplementary Material to: Impact of intensifying nitrogen limitation on ocean net primary production is fingerprinted by nitrogen isotopes

Supplementary Figures

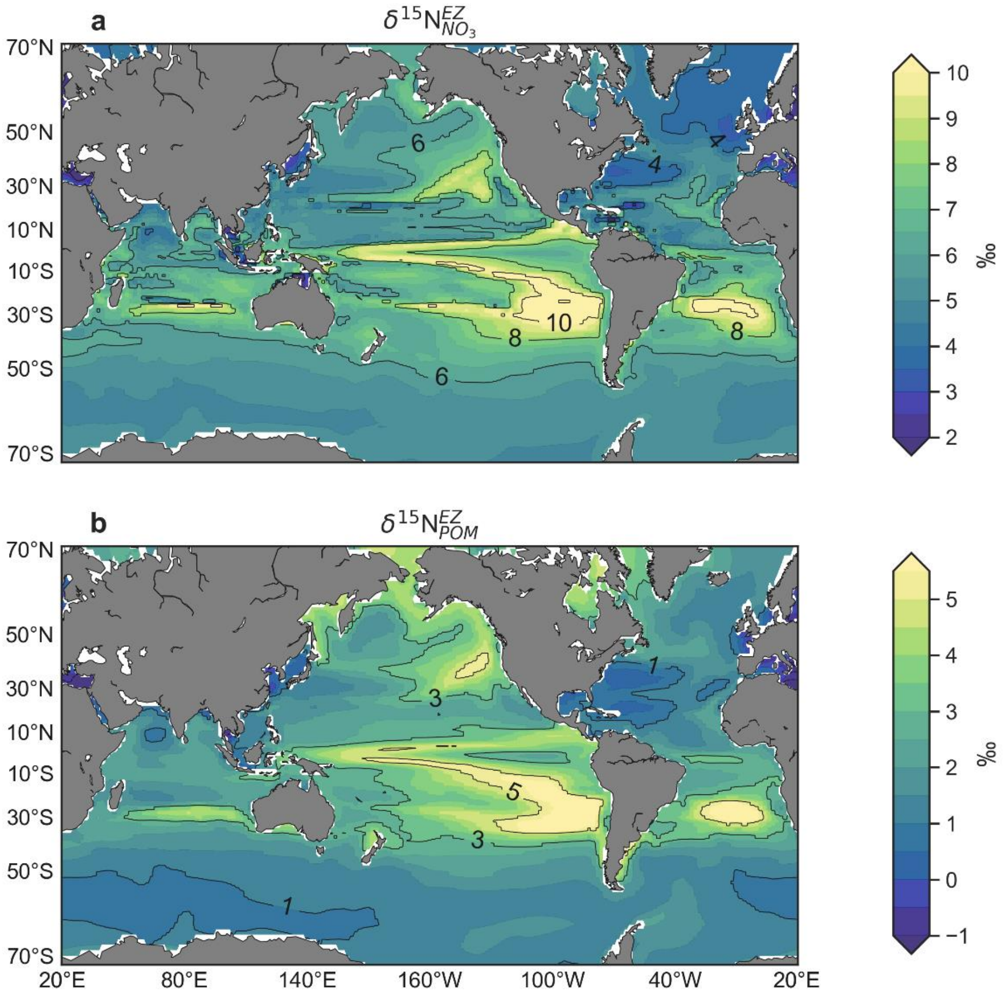

Supplementary Figure 1. Modelled $\delta^{15}N_{NO3}$ (a) and $\delta^{15}N_{POM}$ (b) averaged over the euphotic zone (see Supplementary Figure 7 for depth bounds) and over years 1986-2005 in the full anthropogenic experiment (emissions-driven climate change and anthropogenic nitrogen deposition).

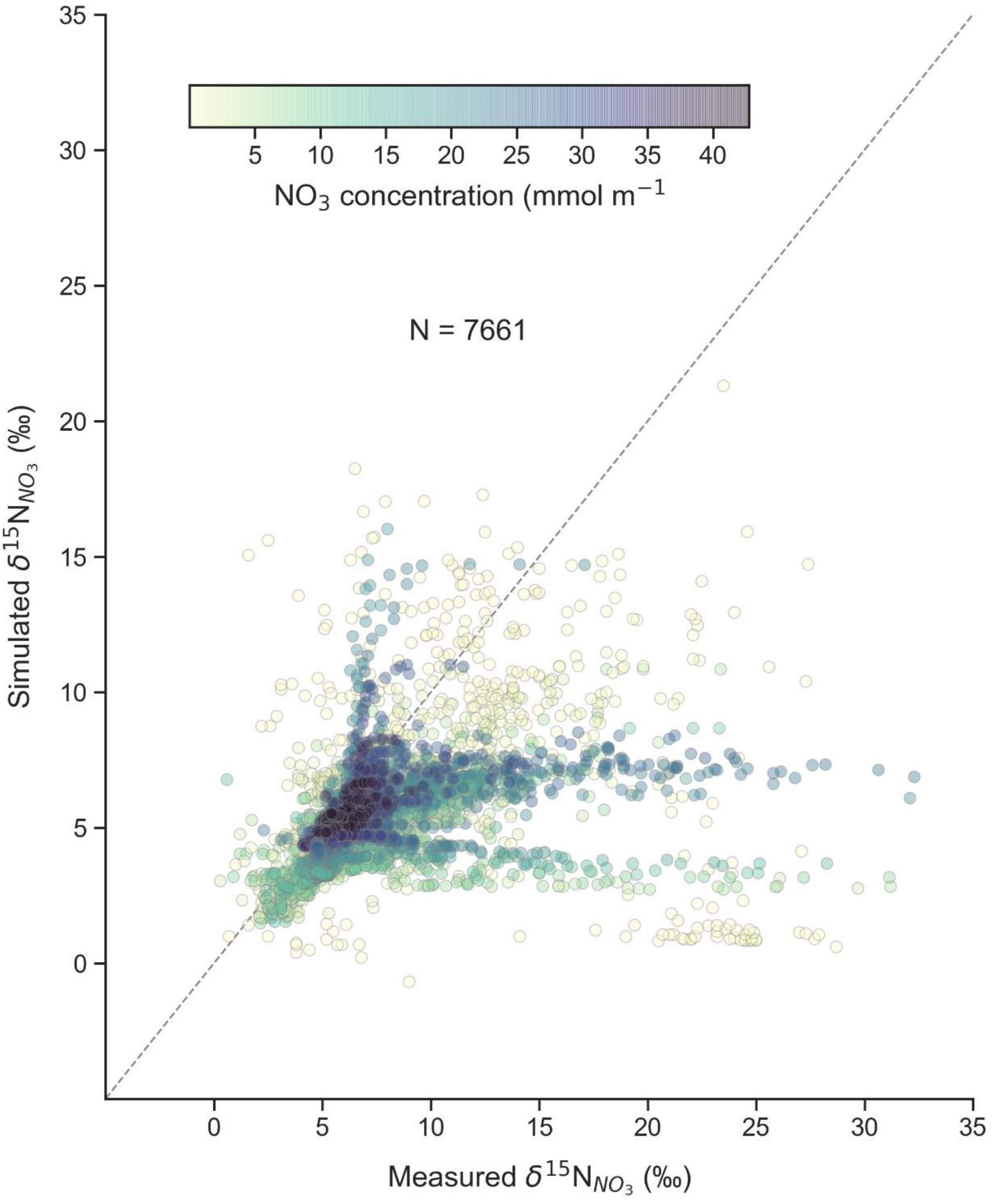

Supplementary Figure 2. Direct comparison of measured and modelled $\delta^{15}N_{NO3}$, coloured by NO_3 concentration. Measurements are a compilation[1] with supplemented data encompassing years 1971-2018. Modelled values are the 1986-2005 average from the climate change experiment with anthropogenic nitrogen deposition.

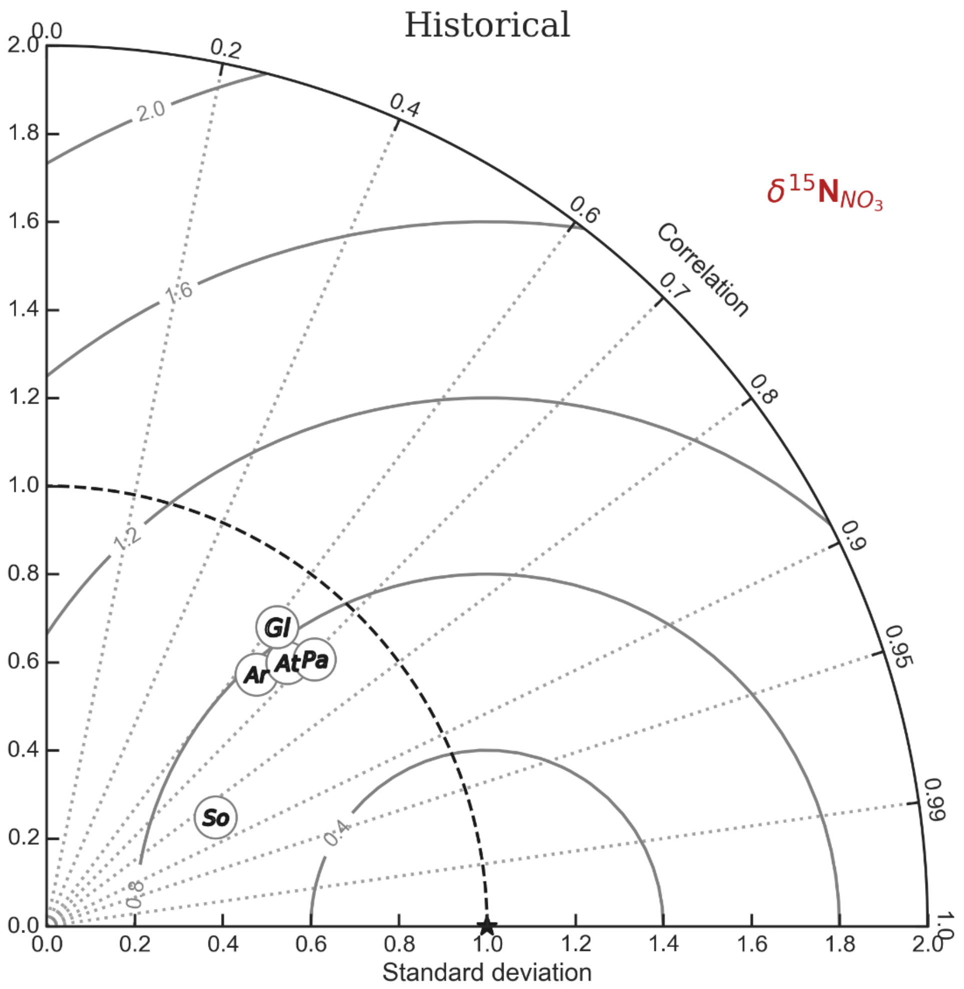

Supplementary Figure 3. Taylor diagram[7] summarising the model-data fit for $\delta^{15}N_{NO3}$. A perfect match between the model and the data would place a marker on top of the star, with a correlation of 1.0, a normalised standard deviation of 1.0, and a root mean square error of 0.0. Correlations (Pearson's r) are represented by radii. Normalised standard deviations are relative to the black dashed line, such that normalised standard deviations less than 1.0 plot below this line. Contours of constant root mean square error are represented by the solid grey lines. Gl = Global; So = Southern Ocean; At = Atlantic; Pa = Pacific; Ar = Arctic. Note that the Indian Ocean does not feature because of a negative correlation.

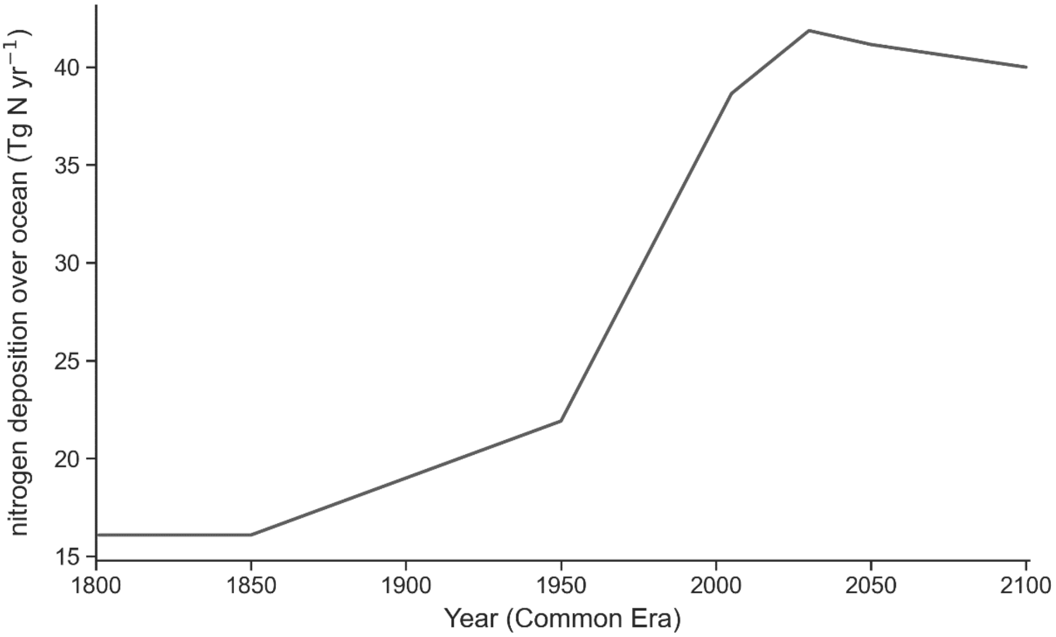

Supplementary Figure 4. Prescribed changes in nitrogen deposition over the ocean from 1801 to 2100.

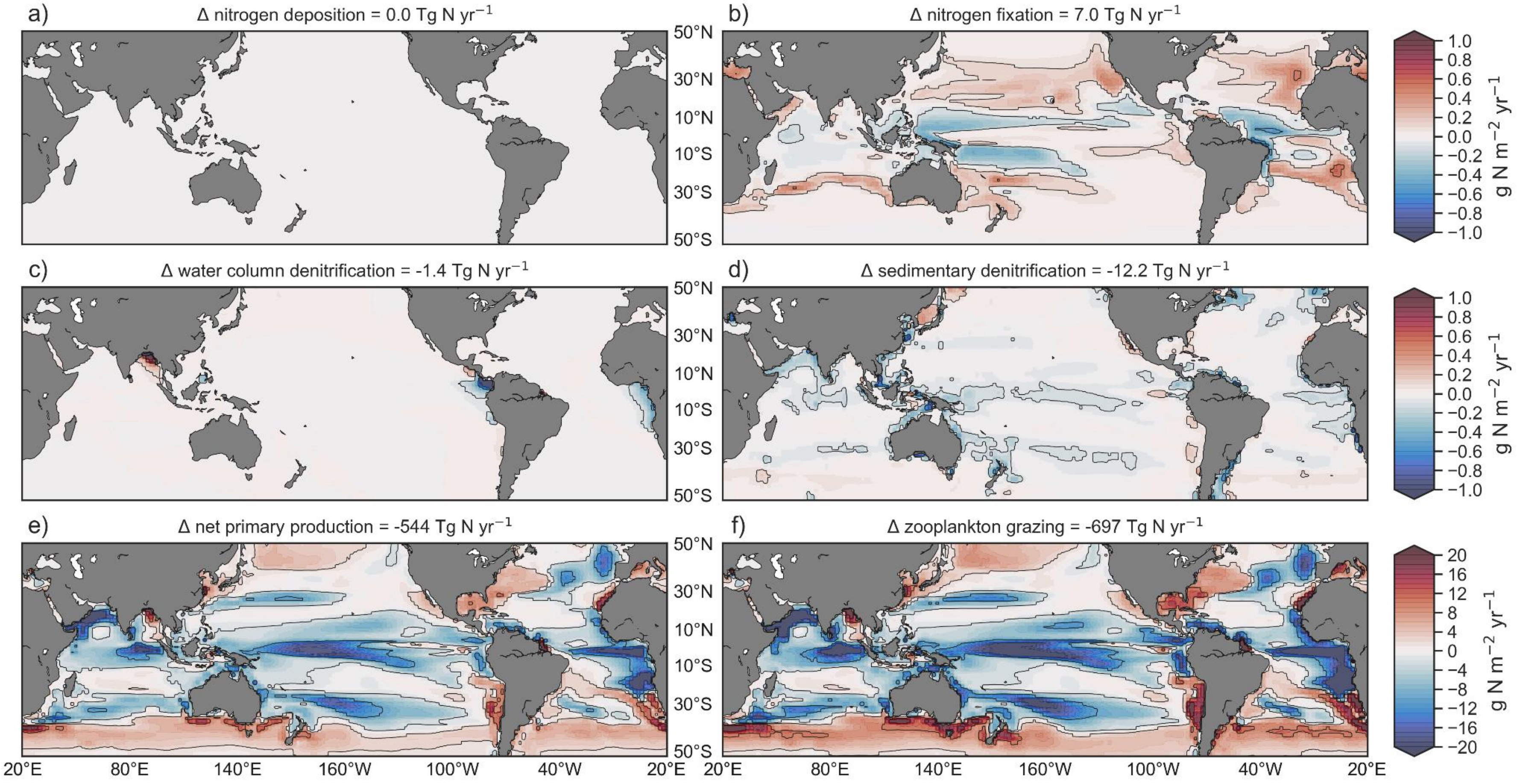

Supplementary Figure 5. Effects of anthropogenic climate change according to RCP8.5 on key marine nitrogen cycle properties. Changes are 2081-2100 relative to the natural experiment. Top row shows major sources: **a**, nitrogen deposition; **b**, nitrogen fixation. Centre row shows major sinks: **c**, water column denitrification; **d**, sedimentary denitrification. Burial of N in sediments is not shown. Bottom row shows internal cycling: **e**, net primary production; **f**, zooplankton grazing. Globally integrated changes are shown above each panel. Note the different scale for the bottom panels.

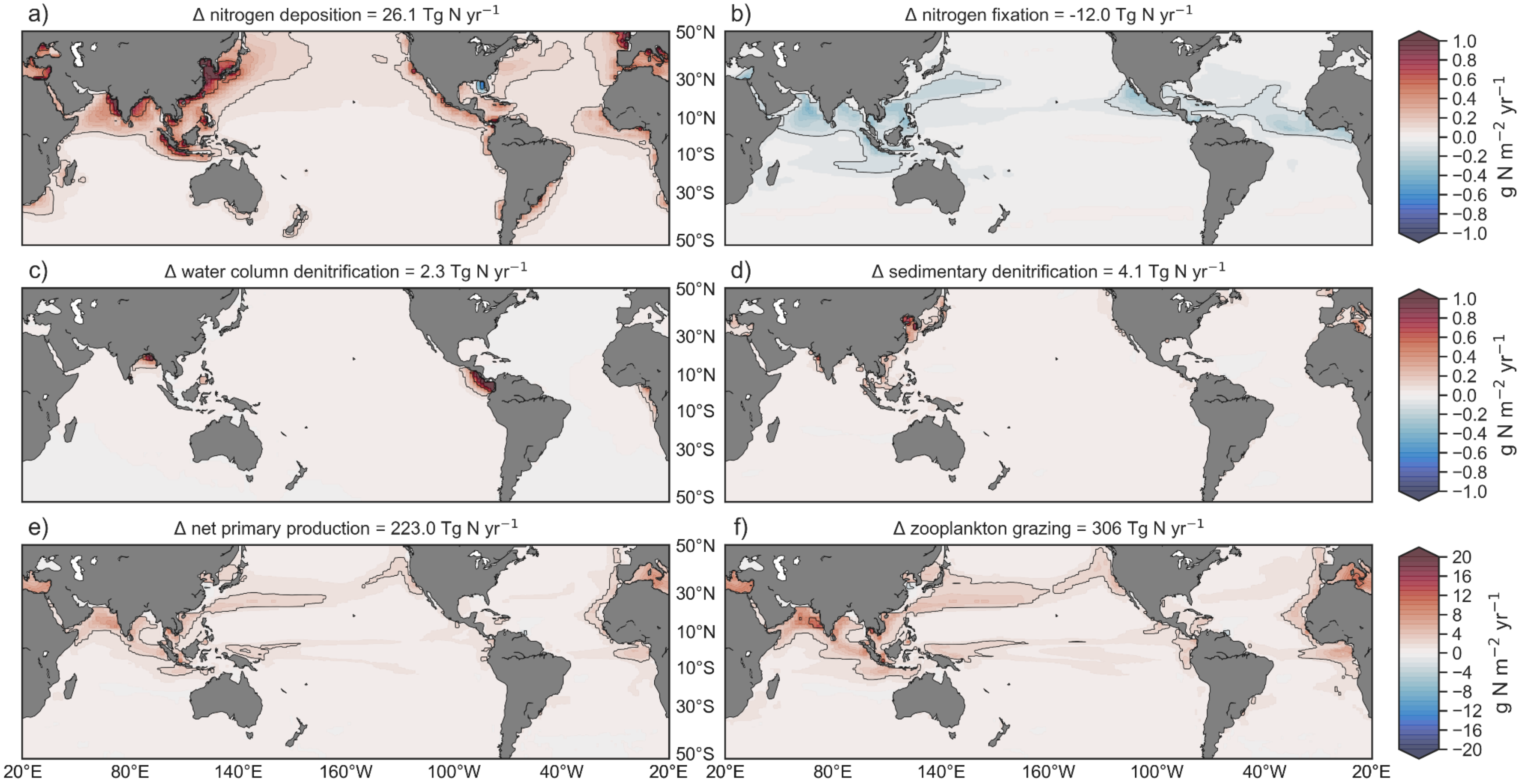

Supplementary Figure 6. Effects of historical rise of nitrogen deposition on key marine nitrogen cycle properties. Changes are 2081-2100 relative to the natural experiment. Top row shows major sources: **a**, nitrogen deposition; **b**, nitrogen fixation. Centre row shows major sinks: **c**, water column denitrification; **d**, sedimentary denitrification. Burial of N in sediments is not shown. Bottom row shows internal cycling: **e**, net primary production; **f**, zooplankton grazing. Globally integrated changes are shown above each panel. Note the different scale for the bottom panels.

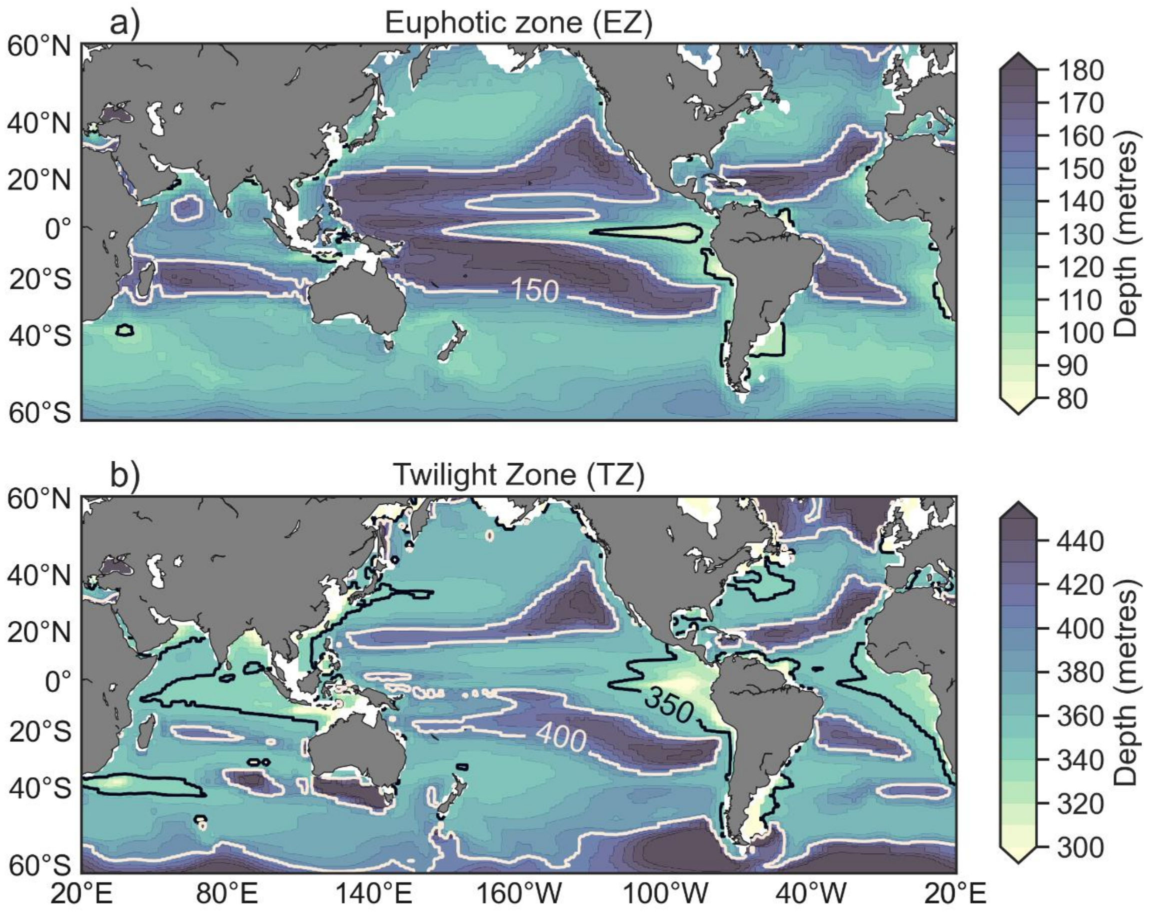

Supplementary Figure 7. Depth boundaries of the euphotic (**a**) and twilight zones (**b**).

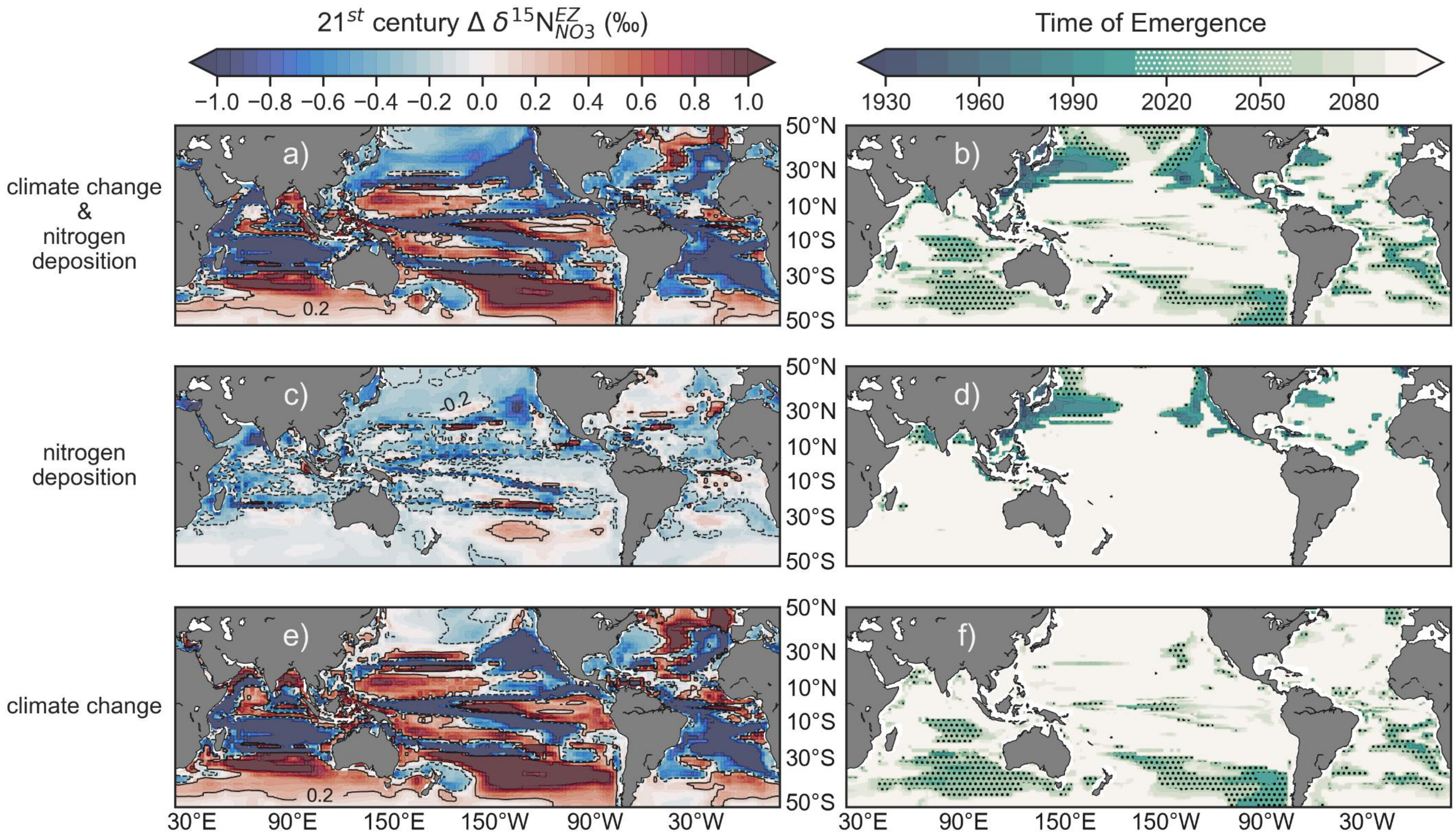

Supplementary Figure 8. Anthropogenic trends of nitrogen isotopes in the euphotic zone and their statistical emergence. **a**, End of 21st century (2081-2100) minus turn of the century (1986-2005) twilight zone $\delta^{15}N_{NO3}$ due to both anthropogenic drivers. **b**, Time of Emergence of twilight zone $\delta^{15}N_{NO3}$. **c**,**d,** same as **a,b,** but for experiment with nitrogen deposition only. **e**,**f**, same as **a,b,** but for experiment with climate change only.

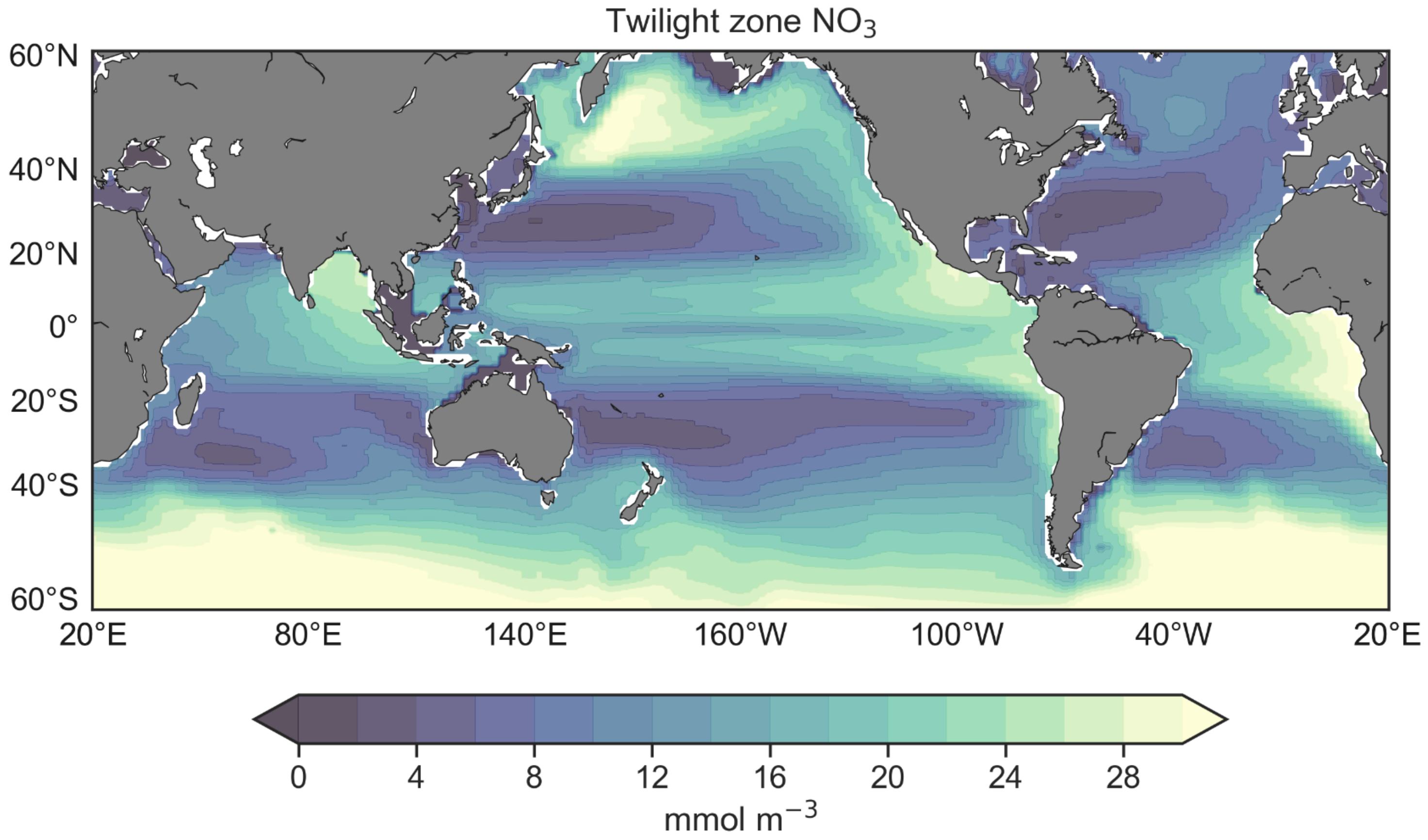

Supplementary Figure 9. Average nitrate (NO_3) concentration within the twilight zone from 1851-2100 under the full anthropogenic simulation (climate change and anthropogenic nitrogen deposition).

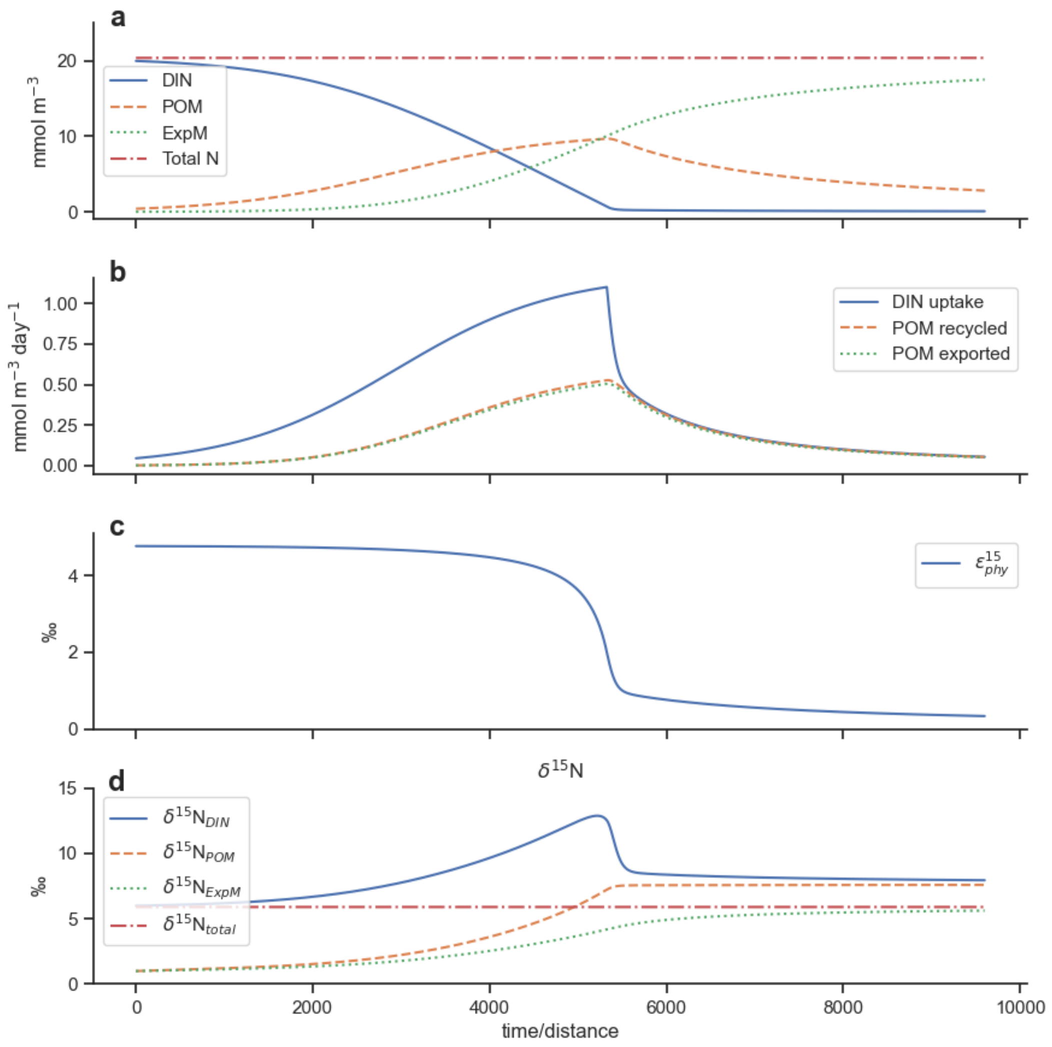

Supplementary Figure 10. Zero-dimensional model following a water parcel within the euphotic zone from point of upwelling in the tropics to nitrogen depletion in the gyre. **a**, Evolution of major pools of dissolved inorganic nitrogen (DIN), particulate organic matter (POM), exported matter (ExpM) and the total concentration of nitrogen, all in units of mmol N m^{-3}. **b**, Rates of DIN uptake, POM recycling and POM export. **c**, Strength of fractionation associated with DIN uptake by phytoplankton. **d**, Accumulated isotopic signatures of the major pools.

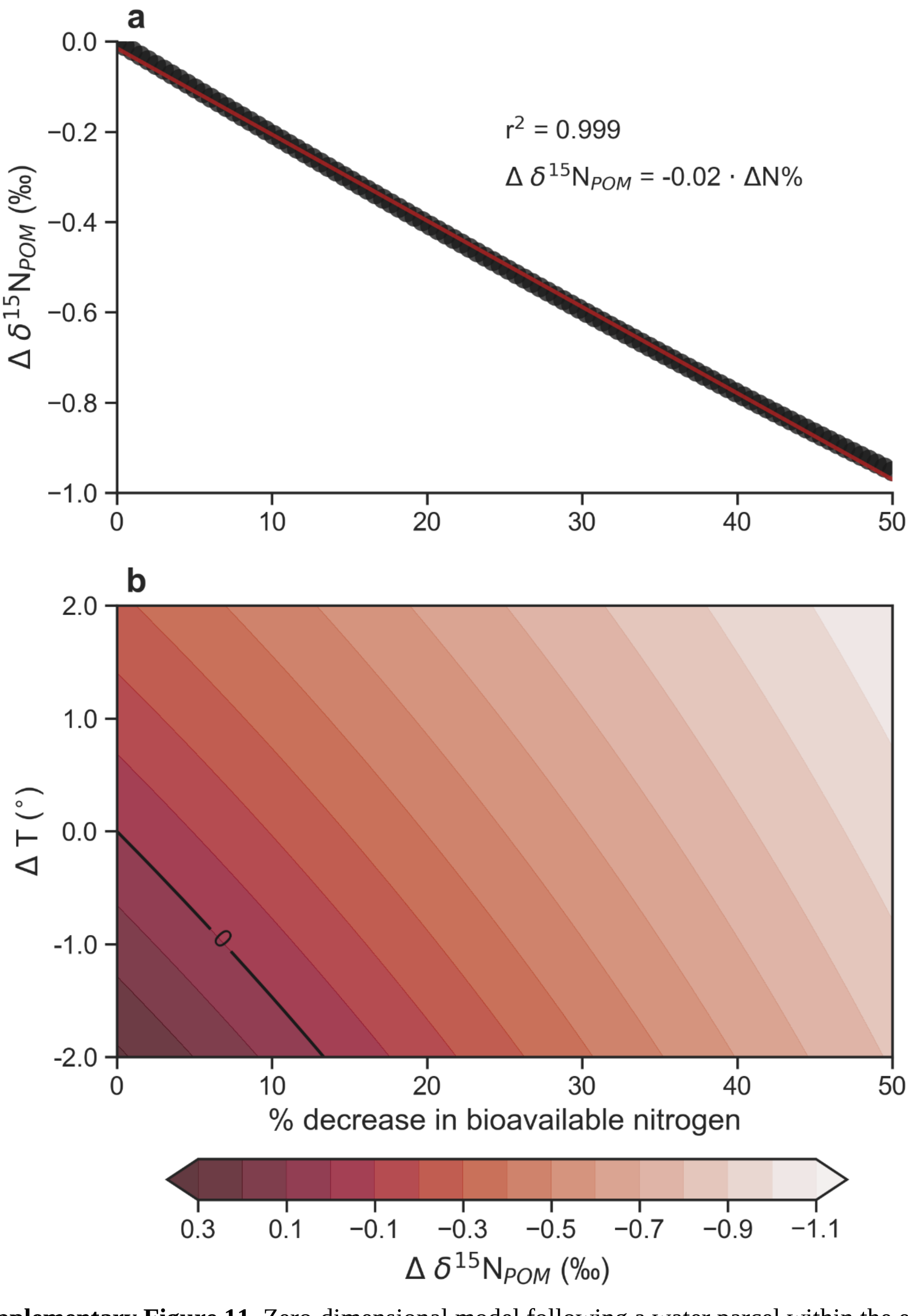

Supplementary Figure 11. Zero-dimensional model following a water parcel within the euphotic zone from point of upwelling in the tropics to nitrogen depletion in the gyre. **a,** Change in final $\delta^{15}N_{POM}$ value following complete utilisation of an initial stock of bioavailable nitrogen concentration (20-10 mmol m^{-3}) given model parameters described in Supplementary Table 1 and constant temperature (i.e. rate of reactions). **b,** effect of changing temperature (16-20 °C), which affects nutrient recycling and growth rates, and initial nitrogen concentration on $\delta^{15}N_{POM}$.

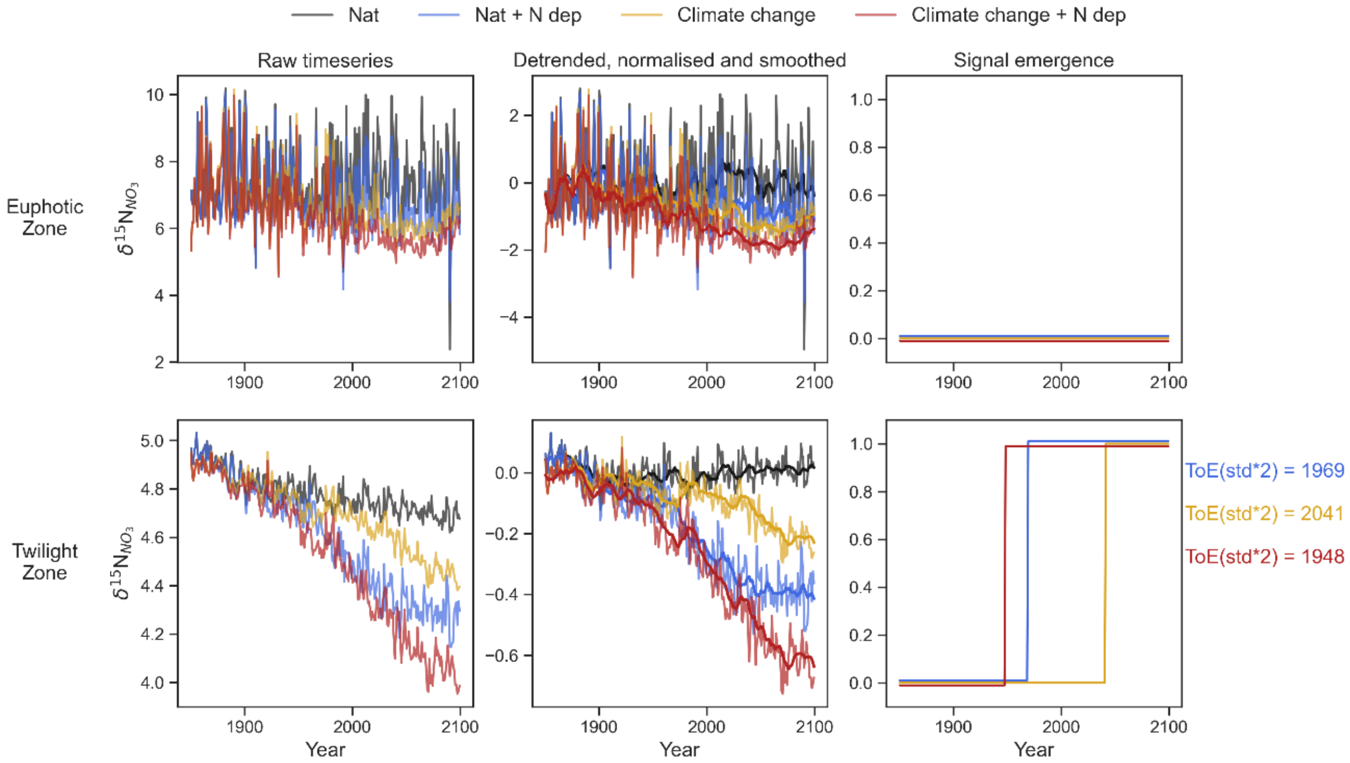

Supplementary Figure 12. Calculating the Time of Emergence at one location in the North Pacific Ocean (180°E, 20°N) in both the euphotic (top) and twilight zones (bottom). Raw timeseries of annual average $\delta^{15}N_{NO3}$ at this location are plotted on the left. These timeseries are detrended and normalised relative to the preindustrial control experiment (Nat) in the centre panels. The resultant timeseries are then smoothed using boxcar smoothing with an 11-year window (thick lines). The emergence of an anthropogenic signal (right panels) occurs when the difference between the smoothed treatment (blue, gold and red lines) and the Nat experiment exceeds the noise of the Nat experiment, which is defined as two standard deviations. Temporary emergences are rejected. For instance, anomalous values of twilight zone $\delta^{15}N_{NO3}$ in under climate change (gold) emerge earlier than 2041, but thereafter return to within the envelope of noise of the Nat experiment. A permanent emergence only occurs at year 2041. Nat = Preindustrial control experiment. N dep = anthropogenic nitrogen deposition. Climate change refers to the anthropogenic climate change associated with historical and future emissions under the Representative Concentration Pathway 8.5[8].

Supplementary Material to: Impact of intensifying nitrogen limitation on ocean net primary production is fingerprinted by nitrogen isotopes

Supplementary Tables

Supplementary Table 1. Constants for zero-dimensional nitrogen isotope model following a water parcel from point of upwelling to residence in the subtropical gyre.

**Variable**	Units	Value	description
maxt	days	100	number of days simulated
dt	day^{-1}	24	timesteps per day
DIN_I	mmol N m^{-3}	20	initial dissolved inorganic nitrogen (DIN) concentration
PON_I	mmol N m^{-3}	$DIN_I \cdot 0.02$	initial phytoplankton concentration
$\delta^{15}N\text{-}DIN_I$	‰	6	initial isotopic signature of DIN
$\delta^{15}N\text{-}PON_I$	‰	1	initial isotopic signature of phytoplankton
ε_{phy}	‰	5	fractionation by phytoplankton
T	°C	18	temperature
T_{growth}	$°C^{-1}$	0.063913	temperature dependent growth coefficient
L_{lim}	unitless	0.15	mean light limitation of phytoplankton in euphotic zone
Fe_{lim}	unitless	0.4	mean iron limitation of phytoplankton in euphotic zone
K_{DIN}	mmol N m^{-3}	1	half-saturation constant for DIN uptake by phytoplankton
P_{mort}	(mmol N m^{-3})$^{-1}$ day^{-1}	0.01	quadratic mortality coefficient for phytoplankton
P_{resp}	day^{-1}	0.01	linear respiration coefficient for phytoplankton
K_{resp}	mmol N m^{-3}	0.2	half-saturation constant for phytoplankton mortality
f_{recmin}	unitless	0.4	minimum fraction of detritus that is recycled
T_{rec}	unitless	0.035	temperature-dependent scaler on recycling

Supplementary References

1. Rafter, P. A., Bagnell, A., Marconi, D. & DeVries, T. Global trends in marine nitrate N isotopes from observations and a neural network-based climatology. *Biogeosciences* **16**, 2617–2633 (2019).
2. Buchanan, P. J., Matear, R. J., Chase, Z., Phipps, S. J. & Bindoff, N. L. Ocean carbon and nitrogen isotopes in CSIRO Mk3L-COAL version 1.0: a tool for palaeoceanographic research. *Geosci. Model Dev.* **12**, 1491–1523 (2019).
3. Karsh, K. L., Granger, J., Kritee, K. & Sigman, D. M. Eukaryotic Assimilatory Nitrate Reductase Fractionates N and O Isotopes with a Ratio near Unity. *Environ. Sci. Technol.* **46**, 5727–5735 (2012).
4. Needoba, J. A., Sigman, D. M. & Harrison, P. J. The mechanism of isotope fractionation during algal nitrate assimilation as illuminated by the $^{15}N/^{14}N$ of intracellular nitrate. *J. Phycol.* **40**, 517–522 (2004).
5. Mariotti, A. *et al.* Experimental determination of nitrogen kinetic isotope fractionation: some principles; ilustration for the denitrification and nitrification process. *Plant Soil* **62**, 413–430 (1981).
6. Sigman, D. M. & Fripiat, F. Nitrogen Isotopes in the Ocean. in *Encyclopedia of Ocean Sciences* 263–278 (Elsevier, 2019). doi:10.1016/B978-0-12-409548-9.11605-7
7. Taylor, K. E. Summarizing multiple aspects of model performance in a single diagram. *J. Geophys. Res.* **106**, 7183 (2001).
8. Riahi, K. *et al.* RCP 8.5—A scenario of comparatively high greenhouse gas emissions. *Clim. Change* **109**, 33–57 (2011).
